# The Effectiveness of Streaming Video on Medical Student Learning: A Case Study

**DOI:** 10.3885/meo.2009.Res00311

**Published:** 2009-08-19

**Authors:** Patrick D. Bridge, Matt Jackson, Leah Robinson

**Affiliations:** Wayne State University School of Medicine, Detroit, USA

**Keywords:** Curriculum delivery, information technology, E-learning, video streaming, LCME Step 1

## Abstract

Information technology helps meet today's medical students’ needs by providing multiple curriculum delivery methods. Video streaming is an e-learning technology that uses the Internet to deliver curriculum while giving the student control of the content's delivery. There have been few studies conducted on the effectiveness of streaming video in medical schools. A 5-year retrospective study was conducted using three groups of students (n = 1736) to determine if the availability of streaming video in Years 1–2 of the basic science curriculum affected overall Step 1 scores for first-time test-takers. The results demonstrated a positive effect on program outcomes as streaming video became more readily available to students. Based on these findings, streaming video technology seems to be a viable tool to complement in-class delivery methods, to accommodate the needs of medical students, and to provide options for meeting the challenges of delivering the undergraduate medical curriculum. Further studies need to be conducted to continue validating the effectiveness of streaming video technology.

Undergraduate medical education (UME) in the 21^st^ century is confronted with many challenges. An increased emphasis on research and clinical practice has resulted in less faculty time for teaching with new demands on administrators in the delivery of medical school curriculum. The projected physician shortage by 2020 provides added pressure for medical schools to increase enrollment,^1^,^2^ creating new challenges. Medical students, on the other hand, are also confronted by their changing environment. Today's need for dual family incomes, as well as increased personal and social demands, warrants an educational environment different from that of the past. Medical schools’ ability to adapt to these internal and external changes and meet students’ needs is vital to the future of UME in the 21^st^ century.

Information technology drives change in healthcare and is an important component in the future of medical education. The first step in meeting today's student's needs is to adapt multiple curriculum delivery methods into the educational environment. Traditional large-group lectures may satisfy the learning and life styles of some students, while others may need more diverse methods. According to Geith and Vignare, on-line and Open Educational Resources are helping to meet the increased demand of non-traditional students.^3^ Offering multiple learning modalities to students not only meets students’ individual needs but also provides medical schools options to meet the challenges facing the delivery of UME.

With the enhancement of technology, e-learning is becoming an educational tool to deliver all aspects of curriculum. E-learning uses the Internet to deliver curriculum content and allows learners to control content delivery, including the sequence, pace, and time.^4^,^5^ E-learning modalities include distance learning and computer-based delivery models. Technology types used for these modalities include web-based platforms, Internet connection tools, streaming video, CDs, video conferencing and other specialized software.^6^

According to Ruiz et al., the use of e-learning has increased over the past 10 years but remains variable in medical schools.^4^ In Lau and Bates’ literature review, 50 articles on the use of e-learning in UME were identified. Of the 50 articles, 33 (66%) focused on Web-based platforms to deliver curriculum, 7 (14%) on specialized software, 5 (10%) on Internet connectivity tools, and 4 (8%) on CDs and video conferencing. Only 1 (2%) research article was found on streaming video.^6^ Studies looking at the effectiveness of on-line learning found no significant differences in grades or retention between on-line learning and face-to-face courses.^3^ Evaluation of e-learning's effectiveness is in its infancy and is dependent on the type of learner and technology used. Students who are motivated, are self regulators, have a specific learning style (e.g., visual) and have computer skills perform well in e-learning environments.^7^ According to Ruiz et al., e-learning is as effective as traditional instructor-led methods such as lectures, while studies in the medical literature demonstrate student satisfaction with e-learning.^4^

Video streaming is an e-learning technology that transmits a continuous video stream over the Internet as digital codes which are then reinterpreted as moving images to a compatible web browser for instant playback.^8^ Video streaming can be synchronous (live) or asynchronous delivery (delayed). Video streaming allows those students attending class to review previous lectures and update notes at their convenience. Students who rely strictly on streaming video can view lectures on their own time, at their own pace, and as frequently as needed. Students can learn efficiently and report satisfaction with a flexible learning experience.^9^

On the other hand, some feel video streaming may take away from the medical school experience, specifically the interaction and cohesiveness of the students. The availability of video can discourage classroom attendance which, in turn, diminishes teacher-student interaction and lowers cohesiveness. Too much flexibility can negatively influence learning as students who are prone to procrastination choose to skip lecture and fall behind in coursework.^10^

While there have been descriptive studies of the benefits and negative aspects of video streaming,^11^ there have been few studies of the effectiveness of video streaming on student learning. Some studies have been conducted on the effectiveness of video lectures that have shown an improvement in grades and satisfaction with a course.^12^,^13^ The literature on interactive videoconferencing varies depending on the application. Some educational researchers have found interactive videoconferencing to be an effective tool in teaching clinical examination objectives to students at remote sites.^14^,^15^ Other studies have demonstrated that video streaming has a neutral effect or no impact,^16^ while others claim it to be as effective as traditional lectures.^17^

The purpose of this study was to determine if the availability of streaming video in the Year 1–2 basic science curriculum affected program outcomes, specifically Step 1 scores for first-time test-takers. Step 1 of the U.S. Medical Licensure Exam was the most suitable instrument to use in a retrospective study since it is required of all US and Canadian LCME accredited medical schools.

## Methods

**Video Streaming at Wayne State University** - In the 2002/2003 academic year, Wayne State University School of Medicine began to develop policies and procedures for video streaming for basic science lectures. The rationale for this initiative was to accommodate adult learning styles and provide a reviewable learning resource for our students. A committee comprising School of Medicine faculty and administrators, in cooperation with the Wayne State University General Counsel, developed a set of policies for streaming video. These policies were in compliance with fair use policy and the TEACH Act of 2002 that defines how institutions must regulate access to the streaming video content.^18^ The committee also developed a release form by which each faculty member grants faculty permission to use the videotaped content of their presentations for educational purposes only.

All of the lecture presentations and demonstrations in the first and second year lecture auditoria were videotaped using a dual camera system controlled from a remote location. One camera captured the image projected on the screen while the other captured the lecturer's image. A picture-in-picture display was seen by the viewer. The recorded presentation was digitized and compressed before uploading to the streaming server as a Windows Media Video (WMV) file. In addition, the server hosted a downloadable audio-only file in MP3 format. A WMV lecture file is approximately 100 MB and an MP3 audio file approximately 10 MB. Students accessed the streaming video files via Lightweight Directory Access Protocol (LDAP) authentication.

**Study Design and Analysis** - With the institutional review board approval, we conducted a retrospective study to determine if the availability and use of streaming video in the Year 1–2 basic science curriculum was associated with overall Step 1 scores for first-time test-takers. Three cohorts of Year 2 students (n = 1736) from 2001–2007 were used in the study. The first cohort (n = 499) during the 2001–2003 academic years did not have access to the streaming video technology. The second cohort (n = 240) from the 2003–2004 academic year was offered streaming video for all Year 2 large-group lectures only. The third cohort (n = 997) from the 2004–2007 academic years was offered streaming video in both Year 1 and Year 2 large-group lectures. Students included in the study had all taken Step 1 for the first time during their cohort year.

To account for academic differences between the groups, the students’ Medical College Admission Test (MCAT) scores (Verbal, Physical, and Biological subscores and Total score) were also analyzed. For the data analysis, means and standard deviations were calculated. Because of the temporal ordering of the groups, we used a two-tailed Jonckheer-Terpstra (J-T) nonparametric test to assess differences between cohorts. This method tests the hypothesis that as you move from the low cohort to the high cohort on the criterion variable the ordered magnitude will increase or decrease. The J-T test is the best choice when the data are not known to be normally distributed, the design is a between-groups design with multiple levels, and a trend is predicted. A p-value < 0.05 was defined as statistically significant.

## Results

We compared students’ MCAT scores to determine if there were academic differences between the groups. Results indicated no statistically significant differences between the groups on any of the four MCAT scores (Table 1).

**Table 1.** Group comparison of MCAT scores and Step 1 scores

	Group 1: No streaming **(n = 499)**	Group 2: Streaming offered only in Year 2 **(n = 240)**	Group 3: Streaming offered in Year 1 and 2 **(n = 997)**	
		
MCAT/Step 1 Variables	Mean/SD	Mean/SD	Mean/SD	Jonckheere-Terpstra Test
MCAT: Verbal	8.94±1.85	8.72±1.84	9.04±1.85	p≤.359
MCAT: Physical Sciences	9.62±1.98	9.44±1.90	9.70±1.85	p≤.837
MCAT: Biological Sciences	9.84±1.81	9.77±1.58	9.93±1.63	p≤.423
MCAT: Total	28.39±4.72	28.01±4.27	28.67±14.18	p≤.645
Step 1 Scores	210±26.46	212±25.43	215±23.79	p≤.000

Step 1 scores were analyzed to determine if the availability and use of streaming video in the Year 1–2 basic science curriculum was associated with the overall Step 1 scores for first-time test-takers. The Jonckheere-Terpstra test statistic showed a statistically significant difference between the groups (p≤.000). Specifically, as shown in Table 1, the mean scores slightly increased from group 1 though group 3, with the mean score for group 1 (no streaming) being 210, group 2 (streaming only Year 2) 212, and group 3 (streaming in Year 1 & 2) 215.

## Discussion

Our school's educational philosophy has been to integrate multiple curriculum delivery methods to accommodate our students’ diverse learning styles. Medical students have the option of attending traditional large-group lectures during Years 1 and 2 or of accessing the streaming video presentation of lectures at any time. In addition to accommodating individual learning styles, streaming video helps the school effectively manage class size without adversely affecting the educational program. The purpose of this study was to determine if the availability of streaming video in the Year 1–2 basic science curriculum was associated with program outcomes, specifically Step 1 scores for first-time test-takers.

Though we found no statistically significant group differences in academic performance on the MCAT scores, we detected a statistically significant difference in Step 1 scores among the cohorts we studied. Specifically, as streaming video was made systematically more readily available, the students performed better on Step 1 on average. To determine if our students’ performance on Step 1 was part of a national trend, we looked at the National Board of Medical Examiners reports on the performance of examinees taking USMLE Step 1. The data showed a similar national trend toward increasingly higher mean scores during the same time period. Specifically, during the first cohort period of our study the national average was 216, the second cohort 216 and the third cohort period 219, a 3 point increase from the first to the third cohort. As previously discussed our increase was from 2–5 points during the same time period.

From a practical perspective the parallel increase in our Step 1 scores and the national Step 1 scores seems insignificant, even though we found a statistically significant result in our data. Consequently, if we take a closer look at our data, the statistically significant differences may be the result of a small treatment effect and large sample size. With the modest outcomes from our study and the parallel increase in our Step 1 scores and the national Step 1 scores, at a minimum it seems that streaming video's effect on program outcomes may be neutral. This therefore provides evidence for its continued use to offer students additional instructional delivery methods to match their learning styles and life styles until further research can be conducted.

Since the implementation of streaming video in our curriculum, student response has also been overwhelmingly positive. In 2008, as part of our LCME accreditation process, a brief survey was conducted of all four medical school classes (n = 1104) to determine students’ use of streaming video. Students were asked, “Of the non-mandatory lectures in Year 1-2, that you did not attend, did you use streaming video”? Of the 552 (50%) responding, 68% stated they always used streaming, 21% often and 7% sometimes. Only 4% stated they rarely or never used streaming video. Streaming server statistics reveal the popularity of the resource: in a one-month period, on average, there were 108,000 one-hour lecture stream hits. That is equivalent to every first and second year medical student accessing the streaming media server 6 times per day.

A criticism from basic science faculty has been that streaming video reduces lecture attendance and creates an environment that diminishes the students’ socialization. Integrating an adequate number of interactive learning experiences with required attendance, such as small group discussion sessions, labs, etc., maintains the medical school experience for students. At our school approximately 60% of the basic sciences curriculum is composed of labs, small group discussion, interactive reviews, patient interviewing, physical skills training, clinic days, and patient panels, all with required attendance. Students have the opportunity to learn from faculty, patients, and their peers at these required activities.

The nature of education research makes it difficult to control for many extraneous variables. While no major curriculum changes occurred during the study's time period and though there were no statistically significant differences in MCAT scores between the groups, other factors may have influenced outcomes. The study demonstrated a positive association with program outcomes or, at a minimum, a neutral effect. It is also evident through our student survey that streaming video meets the needs of many students. However, with the small number of studies conducted on the effectiveness of streaming video, further studies need to be conducted to continue the validation process. Until these studies are conducted, we conclude that streaming video technology seems to be a viable method to complement in-class delivery methods and to meet the needs of today's medical student.
